# Hypergraph partitioning using tensor eigenvalue decomposition

**DOI:** 10.1371/journal.pone.0288457

**Published:** 2023-07-21

**Authors:** Deepak Maurya, Balaraman Ravindran

**Affiliations:** Computer Science and Engineering, Robert Bosch Centre for Data Science and AI, Indian Institute of Technology Madras, Chennai, India; University of Delaware, UNITED STATES

## Abstract

Hypergraphs have gained increasing attention in the machine learning community lately due to their superiority over graphs in capturing *super-dyadic* interactions among entities. In this work, we propose a novel approach for the partitioning of *k*-uniform hypergraphs. Most of the existing methods work by reducing the hypergraph to a graph followed by applying standard graph partitioning algorithms. The reduction step restricts the algorithms to capturing only some weighted pairwise interactions and hence loses essential information about the original hypergraph. We overcome this issue by utilizing *tensor*-based representation of hypergraphs, which enables us to capture actual super-dyadic interactions. We extend the notion of minimum ratio-cut and normalized-cut from graphs to hypergraphs and show that the relaxed optimization problem can be solved using eigenvalue decomposition of the Laplacian tensor. This novel formulation also enables us to remove a hyperedge completely by using the “hyperedge score” metric proposed by us, unlike the existing reduction approaches. We propose a hypergraph partitioning algorithm inspired from spectral graph theory and also derive a tighter upper bound on the minimum positive eigenvalue of even-order hypergraph Laplacian tensor in terms of its conductance, which is utilized in the partitioning algorithm to approximate the normalized cut. The efficacy of the proposed method is demonstrated numerically on synthetic hypergraphs generated by stochastic block model. We also show improvement for the min-cut solution on 2-uniform hypergraphs (graphs) over the standard spectral partitioning algorithm.

## 1 Introduction

In machine learning, interacting systems are often modeled as graphs. In graph modeling, an interacting object is represented as a node, and an edge captures the interaction between a pair of objects. A conventional approach is to quantify the extent of interaction by associating a positive *weight* to the corresponding edge. This graph formulation is further utilized for various standard machine-learning applications in different domains, such as biology [1], VLSI [2], computer vision [3], transport [4], clustering [5], and semi-supervised learning [6]. Learning on graphs has been an active area of research, ranging from spectral graph theory [7] to recently proposed graph neural networks [8]. A graph representation is limited to capturing only *pairwise* interaction, whereas many real-world systems may involve interactions that may be more complex than the simple pairwise formulation [9]. For instance, a collaboration network may involve agents interacting at a group level (also called super-dyadic interactions), which can not be captured by modeling the system as a graph.

Recently, *hypergraphs* have been used to represent and analyze such complex *super-dyadic* relationships. Hypergraphs are generalizations of graphs where an edge could potentially connect multiple nodes. These edges are commonly referred to as *hyperedges*. A *k*-uniform hypergraph refers to the case when all hyperedges are constrained to contain exactly *k* nodes.

Graph partitioning is an interesting problem that involves partitioning the set of nodes in a graph into multiple subsets such that nodes in one subset are more “similar” to each other as compared to nodes in any other subset. Graph partitioning is utilized in various fields such as biology [1], VLSI [2], and computer vision [3]. One of the widely accepted approaches for graph partitioning is minimizing the ratio-cut or normalized-cut [5] objective function using spectra of the graph [7]. Similarly, hypergraph partitioning has been used in a variety of applications in several domains, such as circuit designing [10], image segmentation [11], object segmentation in videos [12], citation networks [13], and semi-supervised learning [14]. In this work, we define the ratio-cut and normalized-cut on hypergraphs and propose a spectral partitioning algorithm.

Existing hypergraph modeling frameworks can be classified into two paradigms, based on whether they reduce the hypergraph to a graph explicitly [15] or implicitly [13, 16]. These reduction based approaches are quite popular in the machine learning community due to the scalability to large datasets [17–19], and provable performance guarantees of graph-based algorithms [20]. Thus most of the existing approaches make use of hypergraph reduction to utilize standard graph-based algorithms, which defeats the motivation behind using hypergraphs. As graphs are limited to capture only dyadic interactions, the reduction-based approaches fail to model the desired super-dyadic relationships.

Ihler et al. [21] show that the reduction-based approaches can not model a hypergraph cut, i.e., the complete removal of a hyperedge from a given hypergraph. After reducing a hypergraph to a graph, partitioning is performed on the graph. During that process, any partitioning algorithm removes some edges from the graph, which is not guaranteed to have any correspondence to the hyperedges in the original hypergraph. Also, note that two or more non-isomorphic hypergraphs may reduce to the same graph. An example for such a case is presented in S1 File. In order to bridge this existing gap, we propose a hypergraph partitioning algorithm in this work, which removes the hyperedges directly without using reduction to the graph. We use the tensor representation of hypergraphs and further the tensor eigenvalue decomposition for hypergraph partitioning. Note that tensor eigenvalue decomposition is NP-hard for general tensors and cannot be approximated unless P = NP [22].

Tensors have gained increasing attention for modeling hypergraphs, primarily in the mathematics community. For instance, Hu et al. [23] extended the fundamental and well-known theorem in spectral graph theory relating cardinality of zero eigenvalue of the Laplacian of a graph to the number of connected components to the uniform hypergraphs. Specifically, they proved that the algebraic multiplicity of zero eigenvalue of a symmetric Laplacian tensor is equal to the sum of the number of even-bipartite connected components and the number of connected components excluding the number of singletons in the given hypergraph. Such insights can not be revealed from the clique reduction methods and its variants [15]. In the machine learning community, tensor representation of hypergraphs has not gained much attention, except for a few works [24, 25]. In this work, we utilize the tensor representation of hypergraphs for detecting densely connected components by extending the notion of ratio-cut and normalized from graphs to hypergraphs [26]. We propose the novel “hyperedge score” that captures the structural variation of multiple nodes in a hyperedge [27, 28]. The key contributions and outline of this work are presented in the following subsection.

### 1.1 Our contributions

We make the following contributions in this work:

- We propose the ratio-cut and normalized cut for k-uniform hypergraphs. Further, we prove that the solution to the minimization of relaxed ratio-cut or normalized cut problem can be obtained from the eigenvector corresponding to the minimum positive eigenvalue of the unnormalized and normalized Laplacian tensor respectively.
- We propose a novel metric termed as “hyperedge score”, which is defined over each existing hyperedge and is a function of the eigenvector corresponding to minimum positive eigenvalue. This hyperedge score metric is used by our partitioning algorithm to remove the hyperedge directly without performing any reduction on hypergraphs [15].
- We also derive a tighter upper bound on the minimum positive eigenvalue of the normalized Laplacian tensor in terms of hypergraph conductance for even order hypergraphs.
- We demonstrate the efficacy of the proposed algorithm on synthetic hypergraphs (k = 2 and k = 4) generated by stochastic block model (SBM).
- We compare the performance on synthetic graphs (2-uniform hypergraphs) generated by SBM. We also report *n*/8 times improvement of ratio-cut over the conventional spectral partitioning for *cockroach graph*, where *n* is the number of nodes.

### 1.2 Outline

The preliminaries of hypergraph notation and tensor representation are covered in Section 2. The proposed hypergraph partitioning algorithm is presented in Section 3. The functioning and efficacy of the proposed algorithm is demonstrated in Section 4 by experiments on synthetic and real hypergraphs. The main manuscript ends with concluding remarks in Section 5. The numerical details of the illustrative examples are presented in the S1 File after references.

## 2 Preliminaries

In this section, we briefly discuss the prevalent approach of representing hypergraphs and their partitioning. A hypergraph *G* is defined as a pair of *G* = (*V*, *E*), where *V* = {*v*_1_, *v*_2_, …, *v*_*n*_} is the set of entities called vertices or nodes and *E* = {*e*_1_, *e*_2_, …, *e*_*m*_} is a set of non-empty subsets of *V* referred to as hyperedges.

The strength of interaction among nodes in the same hyperedge is quantified by the positive weight represented by $w_{e}=\left\{w_{e_{1}},w_{e_{2}},\ldots ,w_{e_{m}}\right\}$.

The vertex-edge incidence matrix is denoted by **H** and has the dimension |*V*| × |*E*|. The entry *h*(*i*, *j*) is defined to be 1 if *v*_*i*_ ∈ *e*_*j*_ and 0 otherwise.

The degree of node *v*_*i*_ is defined by $d_{v_{i}}=\sum_{e_{j}\in E}w_{e_{j}}h\left(i,j\right)$. We can also define two diagonal matrices, **W**, **D**, with the dimension of *m* × *m*, *n* × *n*, containing the hyperedge weights and node degrees respectively. Note that there is no loss of information in this form of representation of hypergraphs until this point. This implies that a unique hypergraph can be constructed for a given incidence matrix.

### 2.1 Reducing a hypergraph to a graph

Now, we discuss the widely-accepted approach for hypergraph reduction in the machine learning community. The fundamental idea is to reduce a hypergraph to graph and subsequently apply standard graph-based algorithms. In this subsection, we briefly discuss the merits and demerits of these approaches and articulate the reasons for choosing the tensor based representation of hypergraphs.

**Definition 1**. *The clique expansion for hypergraph G*(*V*, *E*) *builds a graph G*_*x*_(*V*, *E*_*x*_ ⊆ *V*^2^) *by replacing each hyperedge with the corresponding clique, E*_*x*_ = {(*v*_*i*_, *v*_*j*_): *v*_*i*_, *v*_*j*_ ∈ *e*_*l*_, *e*_*l*_ ∈ *E*} [15]. *The edge weight w*_*x*_(*u*, *v*) *is given by* $w_{x}(u,v)=\sum_{u,v\in e_{l},e_{l}\in E}w(e_{l})$.

The same could be stated in matrix form as

$$\begin{array}{c} A=HWH^{T}-D \end{array}$$ (1)

where **A** represents the adjacency matrix for reduced hypergraph. Another traditional hypergraph reduction approach is star expansion [29]. Most of the other reduction approaches are build on these. Please see [15] and the references therein, for more details.

This reduction step is very convenient as we can now employ any graph algorithms that scale well and come with theoretical guarantees. A natural question arises on the need for these different reduction based approaches. We believe that each of these reduction approaches preserves a few *but* not all hypergraph properties in the reduction step. The preserved hypergraph property may be useful for the end task of learning on hypergraphs. For example, clustering results can be improved on hypergraphs by preserving node degrees during reduction [30].

More often, the reduction step loses vital information about hypergraphs as two different hypergraphs can reduce to the same graph. This can be seen directly from Eq (1) as two distinct hypergraphs having different **H** and **W** can reduce to the same adjacency matrix **A**. An illustrative example of the same is presented in S1 File.

### 2.2 Tensor representation of hypergraphs

In this subsection, we briefly review the tensor-based representation of hypergraphs [31, 32]. A natural representation of hypergraphs is a *k*-order *n*-dimensional tensor A, which consists of *n*^*k*^ entries and is defined by:

$$\begin{array}{c} a_{i_{1}i_{2}\ldots i_{k}}=\{\begin{array}{cc} \frac{w_{e_{j}}}{(k-1)!} & \text{if}\;\left\{i_{1},i_{2},\ldots ,i_{k}\right\}\in E,\;1\leq i_{1},\ldots ,i_{k}\leq n\\ 0 & \text{otherwise} \end{array} \end{array}$$ (2)

It should be noted that A is a “*super-symmetric*” tensor, i.e, $a_{i_{1}i_{2}\ldots i_{k}}=a_{\sigma (i_{1}i_{2}\ldots i_{k})}$, where *σ*(*i*_1_, *i*_2_, …*i*_*k*_) denotes any permutation of the elements in the set {*i*_1_, *i*_2_, …, *i*_*k*_}. The order or mode of the tensor refers to the hyperedge cardinality, which is *k* for A. The degree of all the vertices can be represented by *k*-order *n*-dimensional diagonal tensor D. The Laplacian tensor L is defined as follows:

$$\begin{array}{c} L=D-A \end{array}$$ (3)

An example demonstrating the tensor representation of a 4-uniform hypergraph is presented in S1 File. The normalized Laplacian tensor, denoted by L can also be defined in a similar manner:

$$\begin{array}{c} \ell_{i_{1}i_{2}\ldots i_{k}}=\{\begin{array}{cc} -\frac{w_{e_{j}}}{(k-1)!}\prod_{i_{j}=1}^{k}\frac{1}{\sqrt[{k}]{d_{i_{j}}}} & \text{if}\;\{i_{1},i_{2},\ldots ,i_{k}\}\in E\\ 1 & \text{if}\;i_{1}=i_{2}\ldots =i_{k}=i,\;i=\{1,2,\ldots ,n\}\\ 0 & \text{otherwise} \end{array} \end{array}$$ (4)

For the sake of completeness, we define the tensor eigenvalue decomposition as:

$$\begin{array}{c} Lx^{k-1}=\lambda x,\;\text{such}\;\text{that}\;x^{T}x=1 \end{array}$$ (5)

where $\left(\lambda ,x\right)\in (R,R^{n}\backslash {\left\{0\right\}}^{n})$ is called the Z-eigenpair and $Lx^{k-1}\in R^{n}$, whose *i*^*th*^ component is:

$$\begin{array}{c} {\left[Lx^{k-1}\right]}_{i}=\sum_{i_{k}=1}^{n}\ldots \sum_{i_{3}=1}^{n}\sum_{i_{2}=1}^{n}l_{ii_{2}i_{3}\ldots i_{k}}x_{i_{2}}x_{i_{3}}\ldots x_{i_{k}} \end{array}$$ (6)

The expression for the tensor Laplacian of a hypergraph ($Lx^{k}$) can be computed using the above and $Lx^{k}={\left(Lx^{k-1}\right)}^{T}x$. This is a *k*^*th*^ order polynomial in *n* variables which can be simplified as stated in the following theorem.

**Theorem 2**. *The expression for tensor Laplacian of a hypergraph can be simplified using*

$$\begin{array}{cc} Lx^{k} & =\sum_{i_{1},i_{2},\ldots ,i_{k}=1}^{n}l_{i_{1}i_{2}\ldots i_{k}}x_{i_{1}}x_{i_{2}}\ldots x_{i_{k}}\\ & =\sum_{e_{j}\in E}w_{e_{j}}(\sum_{i_{t}\in e_{j}}x_{i_{t}}^{k}-k\prod_{i_{t}\in e_{j}}x_{i_{t}})=\sum_{e_{j}\in E}w_{e_{j}}k(\underset{i_{t}\in e_{j}}{AM(x_{i_{t}}^{k})}-\underset{i_{t}\in e_{j}}{GM(|x_{i_{t}}{|}^{k})}{\left(-1\right)}^{n_{s,j}}) \end{array}$$ (7)

*where* $n_{s,j}=\left|\left\{i_{t}:x_{i_{t}}<0,i_{t}\in e_{j}\right\}\right|$, *AM and GM stand for arithmetic and geometric means respectively*.

*Proof*.

$$\begin{array}{cc} Lx^{k} & =\sum_{i_{k}=1}^{n}\;\ldots \sum_{i_{2}=1}^{n}\;\sum_{i_{1}=1}^{n}\;\left(d_{i_{1}i_{2}\ldots i_{k}}-a_{i_{1}i_{2}\ldots i_{k}}\right)x_{i_{1}}x_{i_{2}}\ldots x_{i_{k}}\\ & =\sum_{i=1}^{n}\;d\left(v_{i}\right)x_{i}^{k}-\sum_{i_{k}=1}^{n}\;\ldots \sum_{i_{2}=1}^{n}\;\sum_{i_{1}=1}^{n}\;a_{i_{1}i_{2}\ldots i_{k}}x_{i_{1}}x_{i_{2}}\ldots x_{i_{k}}\\ & =\sum_{i=1}^{n}\;\sum_{i_{k}=1}^{n}\;\ldots \sum_{i_{3}=1}^{n}\;\sum_{i_{2}=1}^{n}\;a_{ii_{2}i_{3}\ldots i_{k}}x_{i}^{k}-\sum_{i_{k}=1}^{n}\;\ldots \sum_{i_{2}=1}^{n}\;\sum_{i_{1}=1}^{n}\;a_{i_{1}i_{2}\ldots i_{k}}x_{i_{1}}x_{i_{2}}\ldots x_{i_{k}}\\ & =\sum_{i=1}^{n}\;(\sum_{\left(i_{2},i_{3},\ldots ,i_{k}\right)\in e_{j}}\frac{w_{e_{j}}}{(k-1)!}x_{i}^{k})-(\sum_{\left(i_{1},i_{2},\ldots ,i_{k}\right)\in e_{j}}\frac{w_{e_{j}}}{(k-1)!}x_{i_{1}}x_{i_{2}}\ldots x_{i_{k}}) \end{array}$$ (8)

As there are (*k* − 1)! and *k*! permutations of the first and second term respectively:

$$\begin{array}{cc} Lx^{k} & =\sum_{e_{j}\in E}\;w_{e_{j}}(\sum_{i_{t}\in e_{j}}\;\frac{(k-1)!}{(k-1)!}x_{i_{t}}^{k}-\frac{k!}{(k-1)!}x_{i_{1}}x_{i_{2}}\ldots x_{i_{k}})\\ & =\sum_{e_{j}\in E}\;w_{e_{j}}(\sum_{i_{t}\in e_{j}}\;x_{i_{t}}^{k}-k\prod_{i_{t}\in e_{j}}\;x_{i_{t}})\\ & =\sum_{e_{j}\in E}\;w_{e_{j}}(k\frac{\sum_{i_{t}\in e_{j}}x_{i_{t}}^{k}}{k}-k{\left(\prod_{i_{t}\in e_{j}}\;{\left|x_{i_{t}}\right|}^{k}\right)}^{\frac{1}{k}}{\left(-1\right)}^{n_{s,j}})\\ & =\sum_{e_{j}\in E}\;w_{e_{j}}k(\underset{i_{t}\in e_{j}}{AM(x_{i_{t}}^{k})}\;-\underset{i_{t}\in e_{j}}{GM(|x_{i_{t}}{|}^{k})}\;{\left(-1\right)}^{n_{s,j}}) \end{array}$$ (9)

The above polynomial expression can be viewed as generalization of the graph, as for any edge {*a*, *b*}, the objective function ${\left(x_{a}-x_{b}\right)}^{2}=x_{a}^{2}+x_{b}^{2}-2x_{a}x_{b}$.

**Theorem 3**. *The expression for the normalized tensor Laplacian of a hypergraph*

$$\begin{array}{cc} Lx^{k} & =\sum_{e_{j}\in E}\;w_{e_{j}}(\sum_{i_{t}\in e_{j}}\;\frac{x_{i_{t}}^{k}}{d_{i_{t}}}-k\prod_{i_{t}\in e_{j}}\;\frac{x_{i_{t}}}{\sqrt[{k}]{d_{i_{t}}}})\\ & =\sum_{e_{j}\in E}\;w_{e_{j}}k(\underset{i_{t}\in e_{j}}{AM(\frac{x_{i_{t}}^{k}}{d_{i_{t}}})}-\underset{i_{t}\in e_{j}}{GM({\frac{|x_{i_{t}}|}{d_{i_{t}}}}^{k})}{\left(-1\right)}^{n_{s,j}}) \end{array}$$

*where* $n_{s,j}=\left|\left\{i_{t}:x_{i_{t}}<0,i_{t}\in e_{j}\right\}\right|$.

*Proof*. Similar to Theorem 2

This theorem for hypergraphs will be used in the later sections for proving other theorems. With basics covered in this section, we focus on the main problem of hypergraph partitioning in the next section.

## 3 Partitioning of hypergraphs

We start this section with brief review of spectral graph theory for partitioning of graphs [26] and further propose these ideas for hypergraphs.

### 3.1 Partitioning of graphs

Let the *p* parts of a partition of vertex set *V* be denoted by sets *C*_1_, *C*_2_, …, *C*_*p*_ such that

$$\begin{array}{c} C_{i}\neq \emptyset ,\;C_{i}\subset V,\;\cup_{i=1}^{p}C_{i}=V,\;C_{i}\cap C_{j}=\emptyset ,\;\forall i,j\in \left[p\right],\;\text{and}\;i\neq j \end{array}$$ (10)

The two most commonly used objective function of graph partitioning are Ratio cut [33] and Normalized cut [34]:

$$\begin{array}{cc} \text{Ratio}\;\text{Cut}(C_{1},C_{2},\ldots ,C_{p})= & \sum_{i=1}^{p}\frac{\text{cut}(C_{i},\bar{C_{i}})}{2|C_{i}|},\;\text{where}\;\text{cut}\left(C_{i},\bar{C_{i}}\right)=\sum_{r\in C_{i},s\in \bar{C_{i}}}w_{rs} \end{array}$$ (11)

$$\begin{array}{cc} \text{Normalized}\;\text{Cut}(C_{1},C_{2},\ldots ,C_{p}) & =\sum_{i=1}^{p}\frac{\text{cut}(C_{i},\bar{C_{i}})}{2\text{vol}(C_{i})},\;\text{where}\;\text{vol}\left(C_{i}\right)=\sum_{r\in C_{i}}d_{r} \end{array}$$ (12)

where *w*_*rs*_ denotes the weight of the edge between nodes *r* and *s*, and *d*_*r*_ denotes the degree of *r*^*th*^ node. It is well known that the solution to the relaxed version of minimizing the ratio cut and normalized cut can be obtained from the Fiedler vector of unnormalized and normalized Laplacians, respectively.

The approximation made in the relaxation step is theoretically analyzed [35–37].

### 3.2 Ratio-cut and normalized-cut for hypergraphs

We start the discussion with a formal description of the problem. Let *C*_1_, *C*_2_, …, *C*_*p*_ be the *p* parts of a partition as defined in Eq (10). For a given hypergraph *G*(*V*, *E*, *W*_*e*_), we intend to remove a subset of hyperedges ∂*E* ⊆ *E*, such that *G* \ ∂*E* produces a partition with at least *p* disjoint parts [38, 39]. The hyperedge boundary ∂*E* can be defined as:

$$\begin{array}{c} \partial E=\{e_{j}\in E:e_{j}\cap C_{i}\neq ⌀,e_{j}\cap \bar{C_{i}}\neq ⌀\} \end{array}$$ (13)

for some *i* ∈ [*p*]. It basically denotes the set of hyperedges which “cross” the parts of the partition. The next step is to define the objective function to be minimized for obtaining optimal partitions. The measures described in Eqs (11) and (12) is proposed for graphs and hence not well-suited for hypergraphs as discussed in Example 1 shortly. We propose the following generalization of ratio-cut and normalized-cut for hypergraphs.

**Definition 4**. *The cut cost for the i*^*th*^ *part C*_*i*_ *is denoted by w*_*h*_(*C*_*i*_) *and the total cut cost denoted by w*_*h*,*t*_(*V*) *for all the p parts of a partition is defined as*:

$$\begin{array}{c} w_{h}\left(C_{i}\right)=\sum_{e_{j}\in \partial E}\left|C_{i}\cap e_{j}\right|w_{e_{j}},\;w_{h,t}\left(V\right)=\frac{1}{k}\sum_{i=1}^{p}w_{h}\left(C_{i}\right) \end{array}$$ (14)

The cut cost for a partition and total cut cost defined in Eq (14) reduces to numerator term in Eqs (11) and (12) for *k* = 2 because the term |*C*_*i*_ ∩ *e*_*j*_| reduces to unity ∀*e*_*j*_ ∈ ∂*E* in graphs. We further demonstrate the merits of this cut cost by the following example.

**Fig 1:**
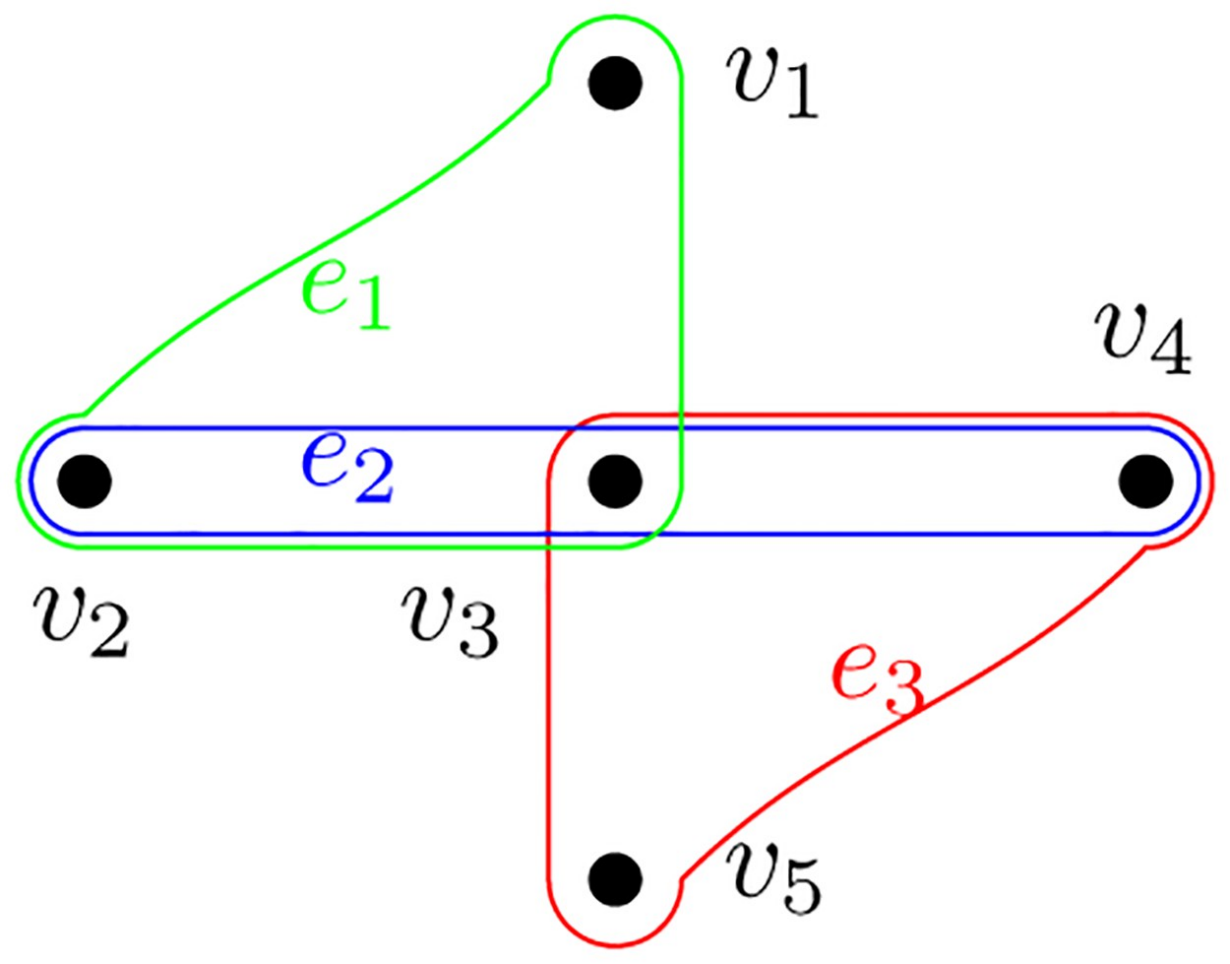
Hypergraph: *H*_1_.

**Example 1**. *Consider the 3-uniform hypergraph shown in* Fig 1. *Consider the partitions obtained after removing hyperedges e*_2_ *and e*_3_. *Let C*_1_ = {*v*_1_, *v*_2_, *v*_3_}, *C*_2_ = {*v*_4_}, *C*_3_ = {*v*_5_}. *The partition cost is given by*

$$\begin{array}{cc} w_{h}\left(C_{1}\right) & =2w_{e_{2}}+w_{e_{3}},\;w_{h}\left(C_{2}\right)=w_{e_{2}}+w_{e_{3}},\;w_{h}\left(C_{3}\right)=w_{e_{3}}\\ w_{h,t}\left(V\right) & =w_{e_{2}}+w_{e_{3}} \end{array}$$

*It should be noted that* $w_{e_{1}}$ *is not reflected in the above cut costs because hyperedge e*_1_ *is not cut. It could be easily verified that this cut cost is not equivalent to clique reduction approach. The cut costs derived for the reduced hypergraph are as follows*:

$$\begin{array}{cc} w_{g}\left(C_{1}\right) & =2\left(w_{e_{2}}+w_{e_{3}}\right),\;w_{g}\left(C_{2}\right)=2\left(w_{e_{2}}+w_{e_{3}}\right),\;w_{g}\left(C_{3}\right)=2w_{e_{3}}\\ w_{h,t}\left(V\right) & =2w_{e_{2}}+3w_{e_{3}} \end{array}$$

*Note that the cut costs derived from both approaches are different. On further inspection, we infer w*_*g*_(*C*_*i*_) = 2*w*_*h*_(*C*_*i*_) *for i* = {2, 3}, *which means the cut cost for partitions C*_2_ *and C*_3_ *in the reduced hypergraph are just a scaled version of costs involved in original hypergraph. The same relation does not hold for partition* *C*_1_ *due to the presence of the term* |*C*_*i*_ ∩ *e*_*j*_| *in* Eq (14). *Please refer to* S1 File *for the computation of these cut costs*.

From this illustrative example, it can be inferred that the proposed cut cost for hypergraphs defined in Eq (14) carries more information about the cut as compared to reduced hypergraphs. The term |*C*_*i*_ ∩ *e*_*j*_| in Eq (14) will lead to a greater penalty for removing hyperedges with more elements from *C*_*i*_. A hyperedge with higher |*C*_*i*_ ∩ *e*_*j*_| is likely to have more association with partition *C*_*i*_, so the corresponding cut should be penalized more.

Minimizing the total cut cost defined in Eq 14 directly may lead to “unbalanced” partitions with minimum cost. To bypass such trivial and undesirable partitions, we propose the normalization.

**Definition 5**. *The Ratio-cut and Normalized-cut for p partitions are defined as*:

$$\begin{array}{cc} Ratio-Cut(C_{1},C_{2},\ldots ,C_{p}) & =\sum_{i=1}^{p}\frac{w_{h}\left(C_{i}\right)}{k|C_{i}{|}^{k/2}} \end{array}$$ (15)

$$\begin{array}{cc} N-Cut(C_{1},C_{2},\ldots ,C_{p}) & =\sum_{i=1}^{p}\frac{w_{h}\left(C_{i}\right)}{k{\left(\text{vol}\left(C_{i}\right)\right)}^{k/2}} \end{array}$$ (16)

where *w*_*h*_(*C*_*i*_) is defined in Eq (14). The above term for ratio-cut and normalized-cut simplifies to Eqs (11) and (12) respectively for *k* = 2. Compared to the similar objective function proposed in literature [13, 19], our objective function normalizes the exponential factor in the denominator. This helps us bypass the partitions with singletons or fewer nodes compared to normalization less than the exponential factor (like linear). Another perspective can be seen from the motivation behind the introduction of |*C*_*i*_| or |*vol*(*C*_*i*_)| term in the denominator of ratio-cut and normalized cut for graphs (*k* = 2). An exponential factor of this normalization factor probably helps us to produce more balanced partitions, which is very much required for hypergraphs. For example, consider a hypergraph with one hyperedge *e*_1_ = {*v*_1_, *v*_2_, *v*_3_} with 3 nodes *v*_1_, *v*_2_, and *v*_3_. Cutting one hyperedge will produce three singletons which we consider as three partitions. A similar definition of normalized associativity can be seen in literature [20, 40].

### 3.3 Hypergraph partitioning algorithm

We wish to find the partition *C*_1_, …, *C*_*p*_ which minimizes the ratio-cut or normalized-cut. It should also be noted that *p* is fixed. For further discussion, we focus on the minimization of ratio-cut, and the same approach can be extended for normalized-cut, as shown later. The optimal partitions can be obtained by solving:

$$\begin{array}{c} \left(C_{1},C_{2},\ldots ,C_{p}\right)=\underset{\left(C_{1},C_{2},\ldots ,C_{p}\right)}{\text{argmin}}\frac{1}{k}\sum_{i=1}^{p}\frac{w_{h}\left(C_{i}\right)}{|C_{i}{|}^{k/2}} \end{array}$$ (17)

Unfortunately, the above problem is NP-hard [26, 41–43]. Inspired from spectral graph theory, we propose to solve a relaxed version of the optimization problem mentioned above.

**Theorem 6**. *The minimization of ratio-cut in* Eq (15) *can be equivalently expressed as*

$$\begin{array}{c} \text{min}\;(\sum_{i=1}^{p}\;Lf_{i}^{k})=\text{min}\;(\sum_{i=1}^{p}\;\sum_{e_{j}\in \partial E}\;\left|C_{i}\cap e_{j}\right|\frac{w_{e_{j}}}{|C_{i}{|}^{k/2}}),\;f_{i,j}=\{\begin{array}{cc} \frac{1}{\sqrt{|C_{j}|}} & \;v_{i}\in C_{j}\\ 0 & otherwise \end{array} \end{array}$$ (18)

*where we define p indicator vectors* **f**_*j*_ *and its i*^th^ *element, denoted by f*_*i*,*j*_ *indicates if the vertex v*_*i*_ *belongs to j*^*th*^ *part of partition, denoted by C*_*j*_. *The solution to the above problem after relaxing* $f_{i}\in R^{n}$ *rather than an indicator vector can be derived from the eigenvector corresponding to the minimum positive eigenvalue stated in* Eq (5).

*Proof*. Given a partition of *p* disjoint sets {*C*_1_, *C*_2_, …, *C*_*p*_}, define the *p* indicator variables **f**_*j*_ = (*f*_1,*j*_, f_2,*j*_, …, *f*_*n,j*_)^⊺^ defined as

$$\begin{array}{c} f_{i,j}=\{\begin{array}{cc} \frac{1}{\sqrt{|C_{j}|}} & \;v_{i}\in C_{j}\\ 0 & \text{otherwise} \end{array} \end{array}$$ (19)

where *i* ∈ [*n*] and *j* ∈ [*p*].

For any part *C*_*i*_, we compute $Lf_{i}^{k}$

$$\begin{array}{c} Lf_{i}^{k}=\sum_{i_{k}=1}^{n}\;\ldots \sum_{i_{2}=1}^{n}\;\sum_{i_{1}=1}^{n}\;l_{i_{1}i_{2}\ldots i_{k}}f_{i_{1},i}f_{i_{2},i}\ldots f_{i_{k},i} \end{array}$$ (20)

We use Theorem 2 to compute the above term

$$\begin{array}{cc} Lf_{i}^{k} & =\sum_{e_{j}\in E}\;w_{e_{j}}\;(\sum_{i_{t}\in e_{j}}\;f_{i_{t},i}^{k}-k\;\prod_{i_{t}\in e_{j}}\;f_{i_{t},i}) \end{array}$$ (21)

There can be three cases for each hyperedge:

1. *e*_*j*_ ⊆ *C*_*i*_: All the nodes in a hyperedge *e*_*j*_ are assigned as $\frac{1}{|C_{i}{|}^{1/2}}$. Both the terms ($\sum_{i_{t}\in e_{j}}f_{i_{t},i}^{k}$ and $k\prod_{i_{t}\in e_{j}}f_{i_{t},i}$) will be $k\frac{1}{|C_{i}{|}^{k/2}}$ and the overall term ($Lf_{i}^{k}$) reduces to 0.
2.
$e_{j}\subseteq {\bar{C}}_{i}$ : All the nodes in hyperedge *e*_*j*_ are assigned 0. Both the terms will be zero and overall term will be zero.
3. *e*_*j*_ ∈ ∂*E*: Some of the nodes are assigned $\frac{1}{\sqrt{|C_{i}|}}$. The second term ($k\prod_{i_{t}\in e_{j}}f_{i_{t},i}$) will be zero and the first term ($\sum_{i_{t}\in e_{j}}f_{i_{t},i}^{k}$) will reduce to $|C_{i}\cap e_{j}|\frac{w_{e_{j}}}{|C_{i}{|}^{k/2}}$.

So the overall term reduce to

$$\begin{array}{c} Lf_{i}^{k}=\sum_{e_{j}\in \partial E}\;w_{e_{j}}\left|C_{i}\cap e_{j}\right|\frac{w_{e_{j}}}{|C_{i}{|}^{k/2}} \end{array}$$ (22)

Summing over the parts, we arrive at

$$\begin{array}{c} \sum_{i=1}^{p}\;Lf_{i}^{k}=\sum_{i=1}^{p}\;\sum_{e_{j}\in \partial E}\;w_{e_{j}}\left|C_{i}\cap e_{j}\right|\frac{w_{e_{j}}}{|C_{i}{|}^{k/2}} \end{array}$$ (23)

The RHS term in Eq (23) is same as the defined ratio cut for hypergraphs (Eq (15)). It should be noted that $f_{i}^{T}f_{i}=1$. As the objective function and constraint are the same under relaxation, the solution to the relaxed optimization problem can be derived from tensor eigenvalue decomposition.

We continue the discussion on partitioning with the following example. Note that a certain ratio-cut approximation is involved while utilizing Theorem 6 for proposing the hypergraph partitioning algorithm using tensor EVD. This approximation is theoretically analyzed in Theorem 8 and Theorem 9.

**Fig 2:**
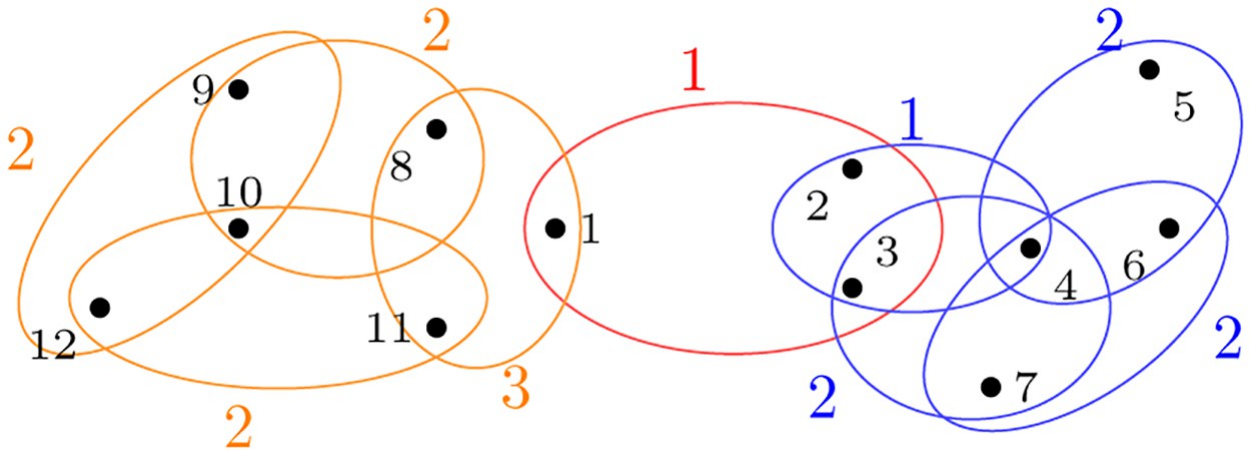
Hypergraph: *H*_2_.

**Example 2**. *Consider the 3-uniform hypergraph shown in* Fig 2. *The colored number indicates the hyperedge weight. It is clear that the optimal partitions are A*_1_ = {2, 3, 4, 5, 6, 7} *and* ${\bar{A}}_{1}$. *The Fiedler eigenvector for this hypergraph is*

$$\begin{array}{c} f^{⋆}=\left[0.33\;0.16\;0.17\;0.13\;-0.05\;0.05\;0.12\;0.39\;0.38\;0.43\;0.39\;0.38\right] \end{array}$$

*A standard approach in spectral graph theory is to use the sign of the elements in the Fiedler vector for partitioning* [26]. *For example, C*_1_ = {*i*|**f**^⋆^(*i*) < 0, *i* ∈ [*n*]}.*Hence, the partitions are C*_1_ = {5} *and* *C*_2_ = *V*\*C*_1_, *which is clearly not optimal*.

From the above example, it is clear that the traditional approach of partitioning does not yield desired partitions for hypergraphs. This is primarily because the eigenvectors of the Laplacian tensor of a hypergraph can not be interpreted in the same way as the eigenvectors of the Laplacian matrix of a graph.

To understand the implication of minimum ratio-cut associated with minimum positive λ^⋆^, we analyze the computation of Laplacian objective function using the Fiedler vector:

$$\begin{array}{c} l_{e_{j}}\left(f^{⋆}\right)=w_{e_{j}}(\sum_{i_{t}\in e_{j}}\;f_{i_{t}}^{k}-k\prod_{i_{t}\in e_{j}}f_{i_{t}}),\;\lambda^{⋆}=\sum_{e_{j}\in E}\;l_{e_{j}}\left(f^{⋆}\right) \end{array}$$ (24)

where $l_{e_{j}}\left(f^{⋆}\right)$ denotes the “score” for hyperedge *e*_*j*_ computed for the eigenvector **f**^**⋆**^. With a slight abuse of terminology, we argue that a higher value of this score indicates the corresponding hyperedges are “close” to separator boundary ∂*E*. The measure of closeness between two nodes is quantified by the minimum number of hyperedges to be traversed for reaching one node to another.

This can be validated easily by careful inspection of hyperedge score $l_{e_{j}}\left(f\right)$, when the vector **f** is treated as the cluster indicator variable shown in Eq (18). The hyperedge score will be non-zero only for the hyperedges on the separating boundary for such ideal choice of **f**. The same can be also interpreted as the score being zero ∀*e*_*j*_ ∈ {*E* \ ∂*E*}. We carry forward the same intuition and prefer to cut the hyperedges with a “higher” score.

The score may not be exactly zero for any hyperedge if the Fiedler vector is used for the score computation as it is obtained for the relaxed minimization of the ratio-cut (Theorem 6). Applying this approach on Example 2, we report a maximum score of 0.017 for the hyperedge {1, 2, 3} and hence cut it to obtain the optimal partitions. It should be noted that we obtain the optimal partitions directly without computing the ratio-cut value *n* − 1 times and taking minimum value like the existing sweep cut-based approaches [33]. The proposed algorithm is summarized in Algorithm 1.

**Algorithm 1**: Hypergraph Partitioning Algorithm

**Result**: Partitions

Construct the tensor Laplacian and derive the Fiedler eigenpair (λ^⋆^, **f**^⋆^).

Calculate the hyperedge score, $l_{e_{j}}\left(f^{⋆}\right)$ by using Eq (24).

**while** number of parts < *p* **do**

 Remove hyperedges with maximum cost (hyepredge score).

**end**

The intuition behind using the hyperedge score for deriving ∂*E* is motivated from spectral graph theory. It is interesting to note that this novel use of hyperedge scores helps to compute a better ratio-cut for the cockroach graph presented in Section 4.

A similar analysis can be performed for the minimization of the normalized cut of hypergraphs.

**Corollary 7**. *The solution to the relaxed optimization problem of minimizing normalized cut mentioned in Proposition 5 can be derived using the eigenvector corresponding to the minimum positive eigenvalue of the normalized Laplacian tensor defined in* Eq (4).

The proof of this corollary is very similar to the proof of Theorem 6. In this case, we choose the indicator variable as

$$\begin{array}{c} f_{i,j}=\{\begin{array}{cc} \frac{1}{\sqrt{\text{vol}(C_{j})}} & \;v_{i}\in C_{j}\\ 0 & \text{otherwise} \end{array} \end{array}$$ (25)

The next step is to compute the $Lx^{k}$, where the normalized Laplacian tensor L is defined in Eq 4. The rest of the proof is very similar to the proof of Theorem 6.

We perform the theoretical analysis of the proposed algorithm and derive an interesting bound on the approximation made in normalized cuts.

**Theorem 8**. *The upper bound on the minimum positive eigenvalue of an even order k-uniform hypergraph is*

$$\begin{array}{c} \lambda_{1}\leq k\phi \left(G\right),\;\phi \left(G\right)=\underset{C\subseteq V}{\text{min}}\;\frac{\sum_{e_{j}\in \partial E}w_{e_{j}}}{\text{min}\;\{vol\left(C\right),\text{vol}\left(\bar{C}\right)\}},\;\text{vol}\left(C\right)=\sum_{i_{j}\in C}d_{i_{j}} \end{array}$$ (26)

*where* λ_1_ *is the smallest eigenvalue satisfying* Eq (5) *for normalized tensor Laplacian* L *and ϕ*(*G*) *refers to the conductance of hypergraph*.

*Proof*. Let **x** be a *n* × 1 vector with $x_{i_{t}}\in \{\frac{\sqrt[{k}]{d_{i_{t}}}}{\omega },\frac{-\sqrt[{k}]{d_{i_{t}}}}{\omega }\}$, where *ω* is defined as

$$\begin{array}{c} \omega ={\left(\sum_{i_{t}=1}^{n}d_{i_{t}}^{\frac{2}{k}}\right)}^{\frac{1}{2}} \end{array}$$

It can be easily verified that **x**^*T*^ **x** = 1. Substitute **x** in the expression for normalized hypergraph Laplacian defined in Eq (4). Please note that the signs of $x_{i_{t}}$ correspond to an arbitrary cut $\left(C,\bar{C}\right)$.

$$\begin{array}{cc} \lambda_{1}\leq Lx^{k} & =\sum_{e_{j}\in E}w_{e_{j}}(\sum_{i_{t}\in e_{j}}\frac{x_{i_{t}}^{k}}{d_{i_{t}}}-k\prod_{i_{t}\in e_{j}}\frac{x_{i_{t}}}{\sqrt[{k}]{d_{i_{t}}}})\\ & =\sum_{e_{j}\in E}w_{e_{j}}(\frac{k-n_{s,j}}{\omega^{k}}+\frac{n_{s,j}{\left(-1\right)}^{k}}{\omega^{k}}-k{\left(-1\right)}^{n_{s,j}}\frac{1}{\omega^{k}}) \end{array}$$ (27)

where $n_{s,j}=\left|\left\{i_{t}:x_{i_{t}}<0,i_{t}\in e_{j}\right\}\right|$. For even order hypergraphs, the above can be reduced to

$$\begin{array}{cc} \lambda_{1} & \leq \sum_{e_{j}\in E}w_{e_{j}}(\frac{k}{\omega^{k}}-k{\left(-1\right)}^{n_{s,j}}\frac{1}{\omega^{k}})\\ & \leq \sum_{e_{j}\in \partial E}w_{e_{j}}(\frac{2k}{\omega^{k}})=\sum_{e_{j}\in \partial S}w_{e_{j}}(\frac{2k}{{\left(\sum_{i=1}^{n}d_{i}^{\frac{2}{k}}\right)}^{\frac{k}{2}}})\\ & \leq \sum_{e_{j}\in \partial E}w_{e_{j}}(\frac{2k}{{\left(\sum_{i=1}^{n}d_{i}\right)}^{\frac{2}{k}\times \frac{k}{2}}})\\ & \leq \sum_{e_{j}\in \partial E}w_{e_{j}}\frac{2k}{2\;\text{min}\;(\text{vol}\left(C\right),\text{vol}\left(\bar{C}\right))}\\ & \leq k\phi (G)\;\;\text{for}\;\text{any}\;C\subseteq V \end{array}$$ (28)

This theorem helps to analyze the order of approximation involved in relaxing the N-min cut problem by deriving the solution through tensor EVD. The tightness of the bound indicates the goodness of the approximation. Several other attempts have been made to derive such approximation bounds for hypergraphs. For example, Chen et al. [27] utilize a different Laplacian tensor and the following hyperedge score to derive similar bound on λ_1_ of a different tensor:

$$\begin{array}{c} l_{e_{j}}\left(x\right)=\sum_{i_{k}\in e_{j}}\;{\left(x_{i_{k}}-\bar{x}\right)}^{k},\;\bar{x}=\frac{1}{k}\sum_{i_{k}\in e_{j}}\;x_{i_{k}},\;\lambda_{1}\leq 2^{k/2}\phi \left(G\right) \end{array}$$ (29)

This is a weaker bound of exponential nature whereas we have proposed tighter bound of linear nature in Theorem 8.

**Theorem 9**. *The upper bound on the minimum positive eigenvalue of the unnormalized Laplacian tensor of an even order k-uniform hypergraph is*:

$$\begin{array}{c} \lambda_{1}\leq k\phi_{r}\left(G\right),\;\phi_{r}\left(G\right)=\underset{C\subseteq V}{\text{min}}\;\frac{\sum_{e_{j}\in \partial E}w_{e_{j}}}{\text{min}\;\{\left|C|,\right|\bar{C}|\}}, \end{array}$$ 30)

*Proof*. Let **x** be *n* × 1 vector with $x_{i_{j}}\in \{\frac{1}{\omega },\frac{-1}{\omega }\}$, where *ω* is defined as

$$\begin{array}{c} \omega ={\left(|V|\right)}^{\frac{1}{2}} \end{array}$$

Please note that **x**^*T*^ **x** = 1. Proceeding in a similar manner to the proof of Theorem 8:

$$\begin{array}{cc} \lambda_{1}\leq Lx^{m} & =\sum_{e_{j}\in E}w_{e_{j}}(\sum_{i_{t}\in e_{j}}x_{i_{t}}^{k}-k\prod_{i_{t}\in e_{j}}x_{i_{t}})\\ & =\sum_{e_{j}\in E}w_{e_{j}}(\frac{k-n_{s,j}}{\omega^{k}}+\frac{n_{s,j}{\left(-1\right)}^{k}}{\omega^{k}}-k{\left(-1\right)}^{n_{s,j}}\frac{1}{\omega^{k}})\\ & \leq \sum_{e_{j}\in \partial E}w_{e_{j}}(\frac{2k}{\omega^{k}})=\sum_{e_{j}\in \partial S}w_{e_{j}}(\frac{2k}{{\left|V\right|}^{k/2}})\\ & \leq \sum_{e_{j}\in \partial E}w_{e_{j}}(\frac{2k}{\left|V\right|})\\ & \leq \sum_{e_{j}\in \partial E}w_{e_{j}}(\frac{2k}{2\;\text{min}\;\left|C|,\right|\bar{C}|})\\ & \leq k\phi (G)\;\;\text{for}\;\text{any}\;C\subseteq V \end{array}$$

where $n_{s,j}=\left|\left\{i_{t}:x_{i_{t}}<0,i_{t}\in e_{j}\right\}\right|$ and *ϕ*_*r*_(*G*) is defined in Eq 30.

It should be noted *ϕ*_*r*_(*G*) defined in Eq 30 is a slightly modified version of the conductance, *ϕ*(*G*) defined in Eq 26. Also, note that *ϕ*_*r*_(*G*) = *d* × *ϕ*(*G*) for *d*-regular hypergraph because *vol*(*C*) = *d* × |*C*| for this particular case. A *d*-regular hypergraph is a hypergraph where each node is constrained to have degree of exactly *d*.

### 3.4 Computation of tensor eigenvectors

The computation of eigenvectors of real super-symmetric tensors is quite challenging and not straightforward as in the case of real symmetric matrices. It is actually NP-hard for general tensors and cannot be approximated unless P = NP [22]. This is primarily due to the non-orthogonality of tensor eigenvectors. There are several other challenging aspects, for example, real symmetric tensors can have complex eigenpairs, unlike the case of matrices. Also, a real symmetric matrix of size *n*×*n* can have a maximum of *n* eigenvalues, whereas a tensor can have much larger number of eigenpairs [31, 44]. Most of the existing works on computation of eigenpairs have been for tensors with special structure [45] or the extreme eigenvalues such as maximum or minimum eigenvalue [46, 47]. As discussed in Section 3.3, only the Fiedler vector is required for partitioning a given hypergraph. As the Fiedler vector is not one of the extreme eigenvectors, the above methods are not helpful for our case.

Recently proposed algorithm to compute all the eigenvalues of a tensor utilizes homotopy methods [48]. They pose the problem as finding the roots of a vector of high order polynomials generated from $P\left(y\right)=Lx^{k-1}-\lambda x=0$, where $y=\left[x\;\lambda \right]\in R^{n+1}$. As it is tough to compute the zeros of *P*(**y**) directly, the core idea of linear homotopy methods is to construct a vector function *H*(**y**, *t*) = (1 − *t*)*Q*(**y**) + *tP*(**y**), where *t* ∈ [0, 1] and *Q*(**y**) is a suitable vector polynomial whose roots can be computed easily. The next step is to slowly iterate from the solution of *H*(**y**, *t* = 0) = *Q*(**y**) = **0** to *H*(**y**, *t* = 1) = *P*(**y**) = **0**. Despite the novel formulation, this approach is forced to compute all the complex eigenpairs even if we are interested in real eigenpairs only.

Before proceeding to the main discussion on the computation of the Fiedler vector, it should be noted that one of the eigenvectors for minimum eigenvalue can be found analytically by exploiting the particular structure of Laplacian tensor [32]. In fact, the minimum eigenvalue of Laplacian tensor is known to be 0, and the corresponding eigenvector is $x=\frac{1}{\sqrt{n}}\left[1\;1\cdots 1\right]$. There can be other eigenvectors for the zero eigenvalue whose graphical implication is discussed in the literature [23]. For our problem, we may use the approach by Cui et al. [49], which computes all the real eigenvalues sequentially from maximum to minimum by using Jacobian semidefinite relaxations in polynomial optimization to avoid computing all eigenvalues.

They formulate the following problem to compute λ_*i*+1_ assuming λ_*i*_ is known:

$$\begin{array}{cc} \text{max} & \;f\left(x\right)=Lx^{k}\\ \text{such}\;\text{that} & \;f\left(x\right)\leq \lambda_{k}-\delta \;\&\;h_{r}\left(x\right)=0,\;\left(r=1,\ldots ,2n-2\right) \end{array}$$ (31)

where 0 < *δ* < λ_*i*_ − λ_*i*+1_ and *h*_*r*_(**x**) is defined as:

$$\begin{array}{c} h_{r}\left(x\right)=\sum_{i+j=r+2}\frac{\partial f(x)}{\partial x_{i}}\frac{\partial g(x)}{\partial x_{j}}-\frac{\partial f(x)}{\partial x_{j}}\frac{\partial g(x)}{\partial x_{i}} \end{array}$$ (32)

where $g\left(x\right)=x_{1}^{2}+x_{2}^{2}+\ldots +x_{n}^{2}-1$ is a normalization constraint. They further utilize Lasserre’s hierarchy of semidefinite relaxations [50] to solve the above problem.

The computation of the objective function *f*(**x**) and the constraints *h*_*r*_(**x**) is expensive and takes *O*(*n*^*k*^) for general tensors. Using Theorem 2, the objective function can be computed in linear time *O*(*m*) for Laplacian tensors. The constraint can also be simplified using:

$$\begin{array}{c} \frac{\partial f(x)}{\partial x_{i}}=\sum_{e_{p}\in E_{i}}kw_{e_{p}}(x_{i}^{k-1}-k\prod_{t\in \{e_{p}\backslash i\}}x_{t}) \end{array}$$ (33)

where *E*_*i*_ = {*e*_*q*_|*i* ∩ *e*_*q*_ ≠ ∅, *e*_*q*_ ∈ *E*}. This approach is very helpful as all the eigenvalues need not be computed for the Fiedler eigenvalue. Hence, these closed form expression for the case of Laplacian tensor can be utilized to reduce the number of function evaluations in optimization methods as compared to general tensors.

### 3.5 Related works

As stated earlier, most of the existing methods utilize hypergraph reductions either implicitly [13, 15] or explicitly or coarsening [10] or scalable heuristic methods [19]. For example, Ghoshdastidar et al. [51] utilize the tensor-based representation of hypergraphs but construct a matrix by concatenating the slices of the tensor. Further, they apply the standard spectral partitioning algorithm on the covariance of that matrix. These variants of hypergraph reduction differ in the method of expanding a hyperedge and produce graphs with different edge weights. The Laplacian objective function (Eq (7)) of any graph is second-order polynomial, which captures weighted interaction among two nodes. A second-order polynomial is insufficient for capturing super-dyadic interaction among multiple nodes (≥3) of a hyperedge. Also, note that multiple hypergraphs may reduce to the same graph.

Hein et al. [52] discuss the incapability of reduction methods in preserving the hyperedge cuts for general hypergraphs. We utilize the Laplacian tensor (Eq (7)) to penalize these multiple cuts differently. Few other recent works try to capture these multiple ways of splitting nodes. For example, Li et al. [53] proposes non-uniform clique expansion and provides quadratic approximation under submodularity constraints of the inhomogeneous cost function. Li et al. [28] extends the notion of *p*-Laplacian from graphs to hypergraphs by introducing the following hyperedge score:

$$\begin{array}{c} l_{e_{j}}\left(x\right)=\underset{i_{k},i_{k}^{{}^{\prime }}\in e_{j}}{\text{max}}\;{\left|x_{i_{k}}-x_{i_{k}^{{}^{\prime }}}\right|}^{p} \end{array}$$ (34)

Ideally, any definition of hyperedge score should capture the non-uniformity among the nodes in a hyperedge, but the above equation fails to capture the variation perfectly. For example, consider two hyperedges with cardinality 4 and node labels assigned as {0, 1, 1, 2} and {0, 1, 2, 2}. Eq (34) computes the maximum difference and hence will not differentiate among these two hyperedges but the AM-GM difference (Eq (24)) will capture the variation among all the nodes of the hyperedge. Various other similar formulation of the hyperedge score function are considered in literature [54–56].

## 4 Experiments

We compare the proposed algorithm and sign-based Fiedler vector partitioning on cockroach graphs. Further, the proposed algorithm is examined on synthetic graphs and hypergraphs generated by Erdős Rényi Model [57] and Stochastic Block Model (SBM) [58]. The numerical details of Fiedler vector and hyperedge scores are presented in S1 File.

### 4.1 Proposed algorithm vs sign-based partitioning on cockroach graph

**Fig 3:**
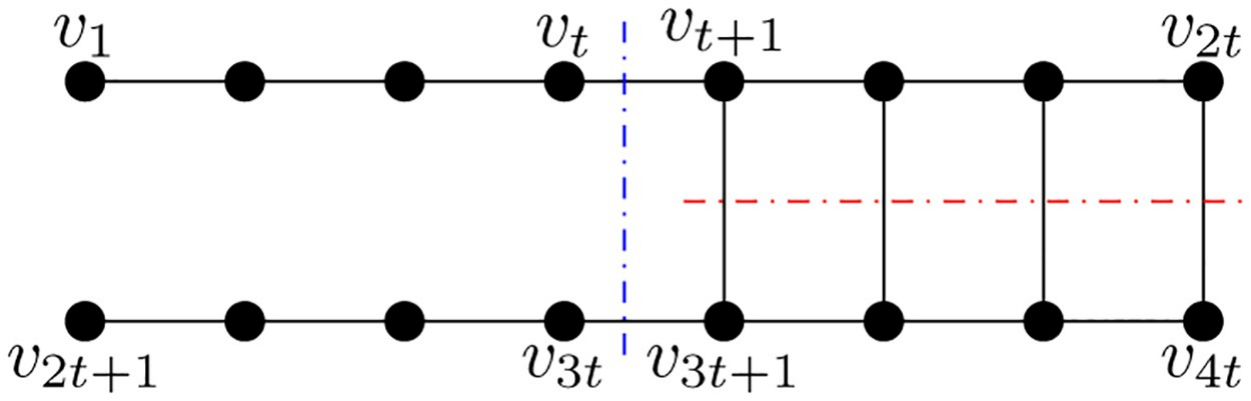
Cockroach graph.

Consider the cockroach graph with 4*t* nodes, as shown in Fig 3. Von Luxburg [26] shows that the conventional sign-based Fiedler vector partitioning does not produce the optimal ratio-cut for cockroach graph. In this example, we show that the proposed algorithm performs better. For comparing the proposed method and sign-based Fiedler vector partitioning, we compute the ratio-cut value by these algorithms.

The analysis shown in this example is valid for general *t* but we have presented the numerical details for *t* = 3. The Fiedler vector for this graph (*t* = 3) is given by:

$$\begin{array}{c} f={\left[\begin{array}{cccccccccccc} -0.49 & -0.41 & -0.26 & -0.07 & -0.02 & -0.01 & 0.49 & 0.41 & 0.26 & 0.07 & 0.02 & 0.01 \end{array}\right]}^{T} \end{array}$$ (35)

So the partitions defined based on the sign of elements in **v** are *A*_1_ = {*v*_1_, *v*_2_, *v*_3_, *v*_4_, *v*_5_, *v*_6_} and $A_{2}=\bar{A_{1}}$. So, the ratioCut $\left(A_{1},\bar{A_{1}}\right)=\frac{3}{6}+\frac{3}{6}=1$.

The next step is to apply the proposed algorithm. The edge score computed from the proposed algorithm are presented in Table 1. The edges {*v*_3_, *v*_4_} and {*v*_9_, *v*_10_} are removed as they are of maximum edge score of 0.0371. So the partitions are $B_{1}=\left\{v_{1},v_{2},v_{3},v_{7},v_{8},v_{9}\right\},B_{2}=\bar{B_{1}}$. Therefore, ratioCut $\left(B_{1},\bar{B_{1}}\right)=\frac{2}{6}+\frac{2}{6}=0.66$. It can be clearly observed that the partitions obtained from the proposed algorithm have a lower ratioCut value compared to the existing method.

**Table 1:** Edge-score for graph in Example 4.1.

Edge	Score
{*v*_3_, *v*_4_}	0.0371
{*v*_9_, *v*_10_}	0.0371
{*v*_4_, *v*_10_}	0.0228
{*v*_2_, *v*_3_}	0.0228
{*v*_8_, *v*_9_}	0.0222
{*v*_1_, *v*_2_}	0.0222
{*v*_7_, *v*_8_}	0.0066
{*v*_4_, *v*_5_}	0.0066
{*v*_10_, *v*_11_}	0.0029
{*v*_5_, *v*_11_}	0.0029
{*v*_6_, *v*_12_}	0.0002
{*v*_11_, *v*_12_}	0.0002
{*v*_5_, *v*_6_}	0.0002

In general, the traditional spectral partitioning makes the red cut shown in the graph and the partition is *A*_1_ = {*v*_1_, …, *v*_2*t*_} and the ratio-cut $\left(A_{1},\bar{A_{1}}\right)=\frac{t}{2t}+\frac{t}{2t}=1$. We utilize the edge scores as suggested in the proposed algorithm and report that the edges {*v*_*t*_, *v*_*t*+1_} and {*v*_3*t*_, *v*_3*t*+1_} have maximum scores. On cutting these edges, the obtained partition is *B*_1_ = {*v*_1_, *v*_2_, …, *v*_*t*_, *v*_2*t*+1_, …, *v*_3*t*_} and hence the ratio-cut $\left(B_{1},\bar{B_{1}}\right)=\frac{2}{2t}+\frac{2}{2t}=\frac{2}{t}$. Therefore, the solution obtained by proposed algorithm is *t*/2 times better than the traditional approach. We have verified it numerically for *t* = {3, 4, …, 50}.

### 4.2 Proposed algorithm vs sign-based partitioning on synthetic graphs & hypergraphs

In this example, we consider different types of synthetic graphs and compare the ratio-cut values computed by the existing and proposed methods. We define the following metric, termed as percentage improvement (PI) to showcase the proposed algorithm’s performance:

$$\begin{array}{c} \text{PI}=\frac{\left(R_{f}-R_{p}\right)}{R_{f}}\times 100 \end{array}$$ (36)

where *R*_*f*_, *R*_*p*_ denotes the ratio-cut value by sign based Fiedler partitioning and proposed algorithm, respectively. A positive value of PI indicates the proposed algorithm has produced a better ratio-cut value and the magnitude of the value represents the extent of the improvement.

#### 4.2.1 Proposed algorithm vs sign-based partitioning on graphs generated by ER model

**Fig 4:**
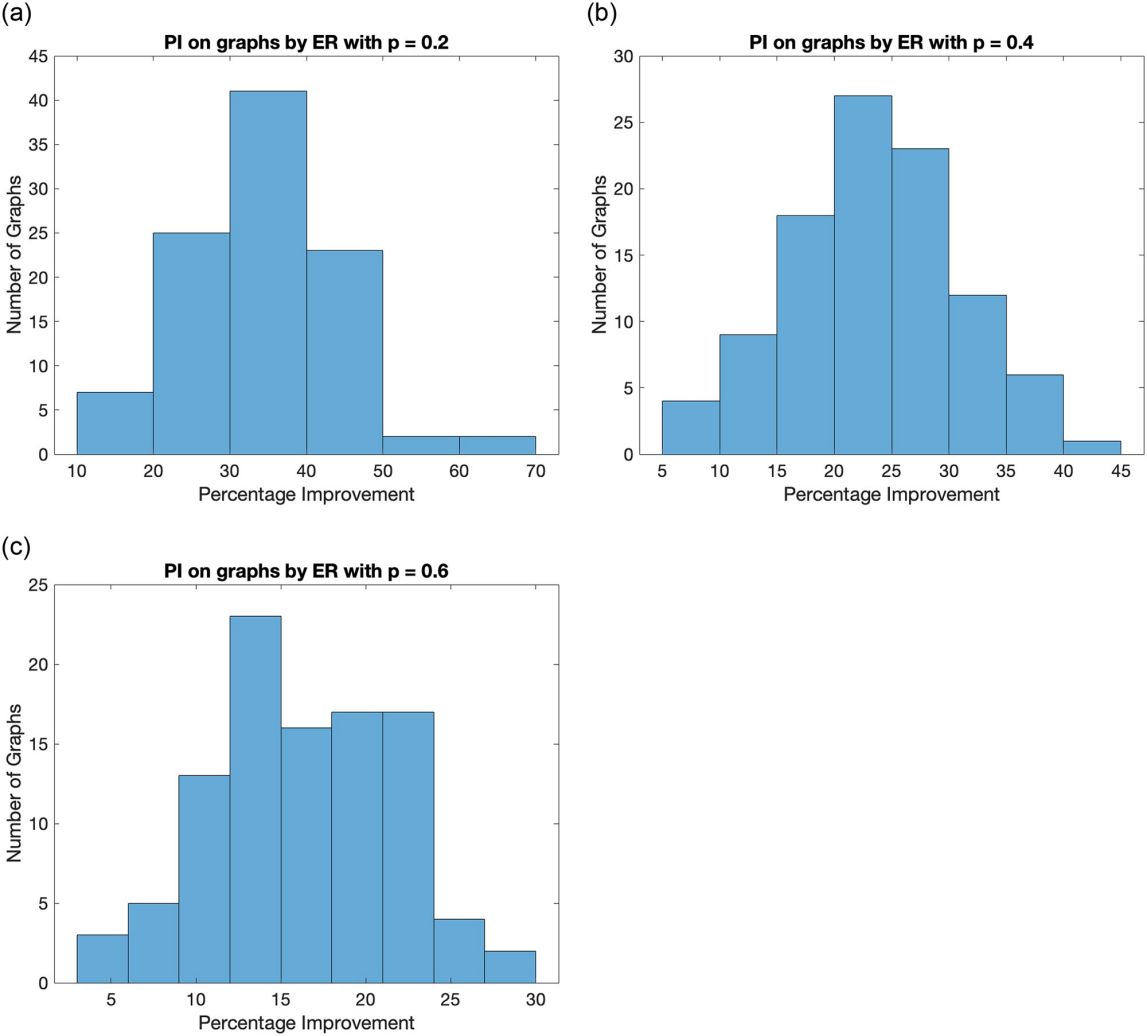
Histogram plot for percentage improvement on ratio-cut value by the proposed method for graphs generated by the ER model for different values of *p*. It shows that the proposed algorithm performs better for all the generated graphs.

We begin with the study on random graphs generated from the Erdős Rényi Model [57] denoted by *G*(*n*, *p*), where *n* is the number of nodes and *p* is the probability of an edge between any two nodes. We compare the ratio-cut values 2 partitions values on 100 different graphs for *n* = 100 and for each value of *p* = {0.2, 0.4, 0.6}. Fig 4 shows the result as a histogram for different values of *p* = {0.2, 0.4, 0.6}. It can be seen that the proposed algorithm performs better than the sign based Fiedler partitioning in all cases.

#### 4.2.2 Proposed algorithm vs sign-based partitioning on graphs generated by SBM

We perform a similar analysis for another graph generation model, referred to as the stochastic block model (SBM). This model provides us the freedom to control the number of parts, the number of nodes in each part (denoted by *n*_1_, *n*_2_), the probability of an edge within a part (denoted by *p*), and across the partition (*q*). Note that *p* = *q* yields ER model with *n* = *n*_1_ + *n*_2_ as discussed previously.

**Fig 5:**
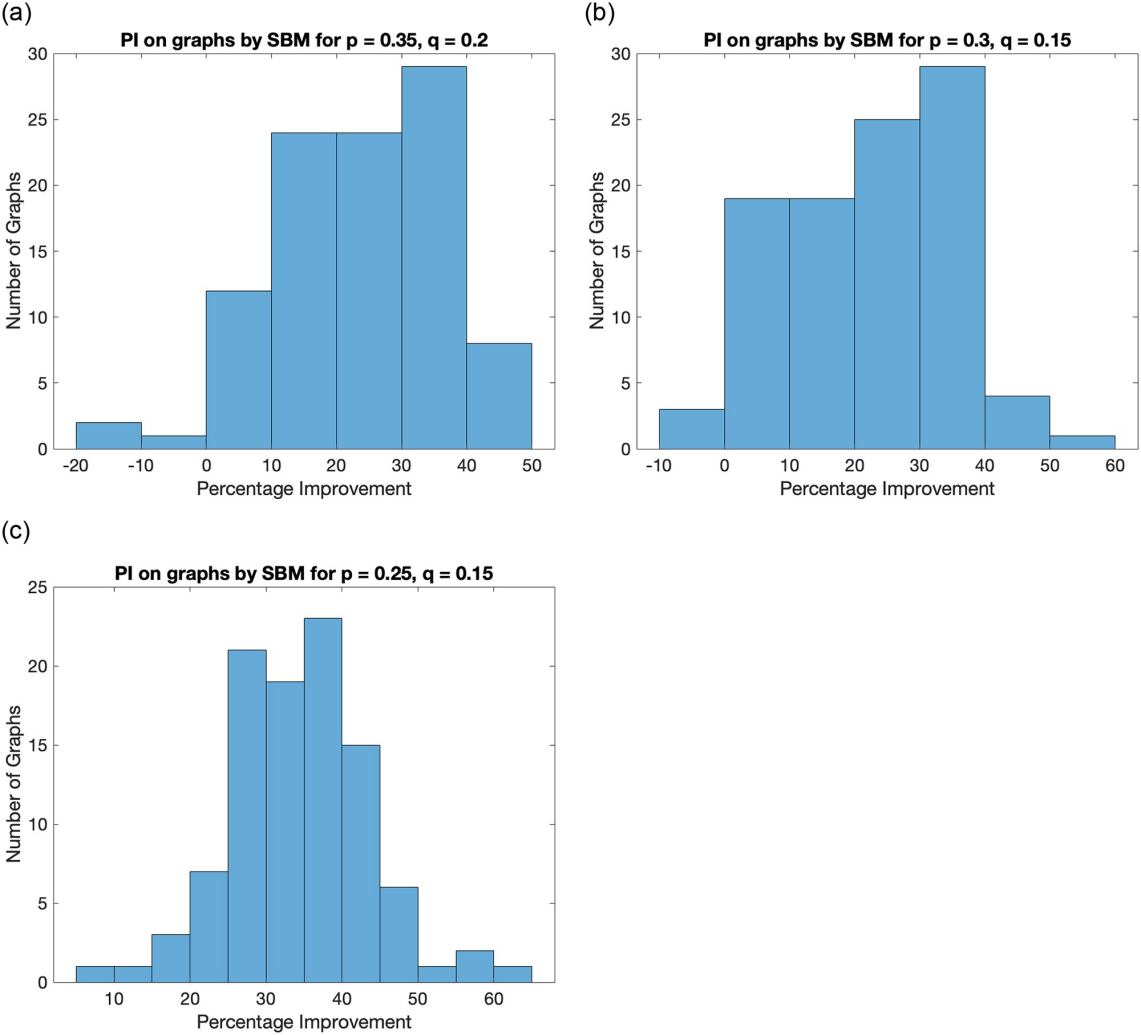
Histogram plot for percentage improvement on ratio-cut value by the proposed method for graphs generated by the SBM for different values of intra-cluster probability (p) and inter-cluster probability (q). It confirms that the proposed algorithm performs better for most of the generated graphs as there are very few cases of negative PI.

We consider the graphs for multiple combinations of probabilities *p*, *q* and 2 partitions with *n*_1_ = *n*_2_ = 50. It should be noted that we consider the SBM with assortative community structure, which implies *p* > *q*. We generate 100 random graphs for each of these settings and compare the ratio-cut values. A histogram plot summarizing the results is presented in Fig 5.

It is evident from Fig 5 that the proposed algorithm produces a lower ratio-cut value for most of the graphs generated by SBM.

#### 4.2.3 Proposed algorithm vs sign-based partitioning on hypergraphs generated by SBM

We perform a similar analysis on synthetic hypergraphs generated by SBM [51]. We generate 100 random 4-uniform hypergraphs with 2 partitions, 60 nodes, and relatively small values of intra-cluster probability (*p*) and inter-cluster probability (*q*) as compared to the case of graphs. This is primarily because the number of possible hyperedges for a 4–uniform hypergraph is (n4), which is much larger than (n2) as compared to the case of graphs.

**Fig 6:**
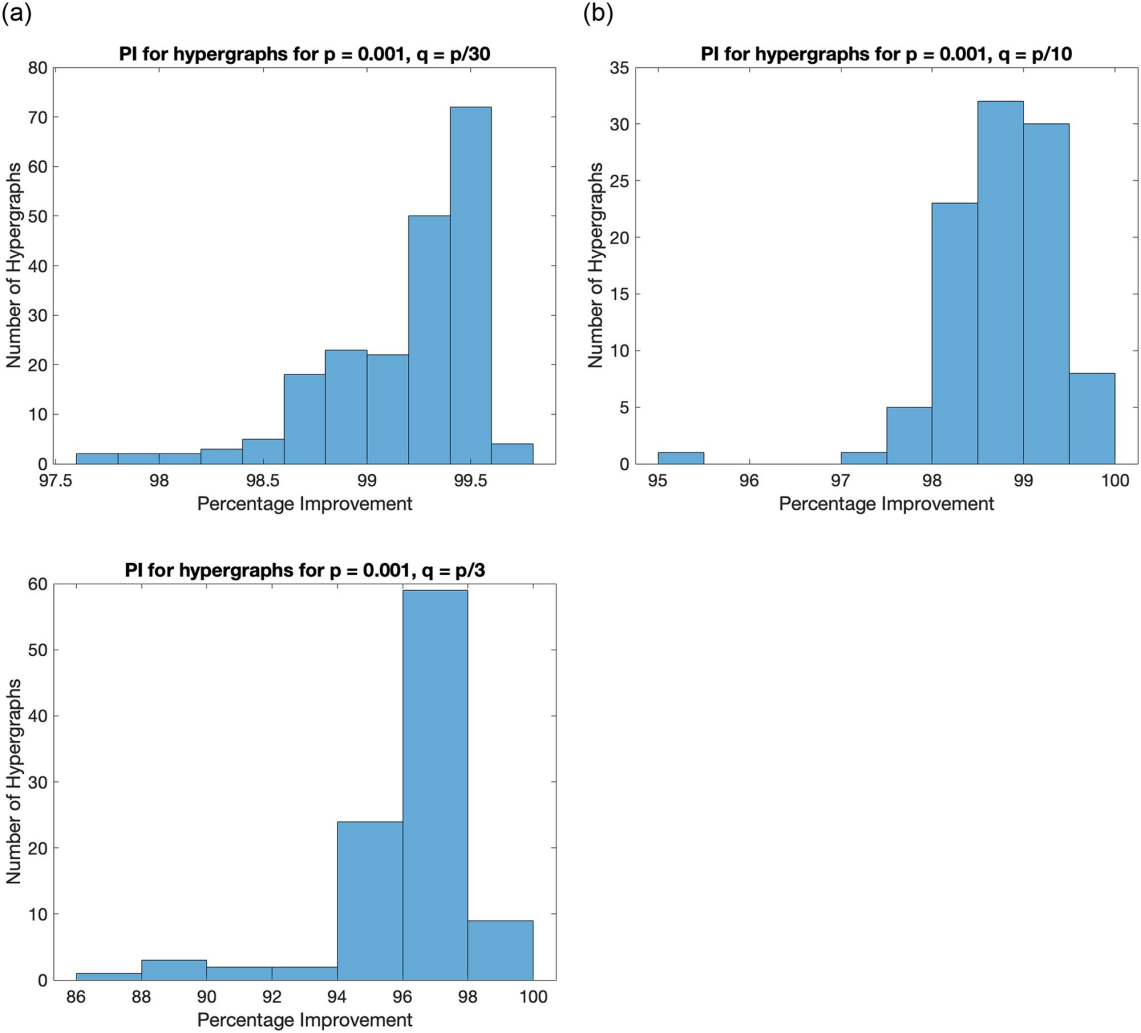
Histogram plot for percentage improvement on ratio cut value by the proposed method for hypergraphs generated by the SBM for different values of *q*. It shows that the proposed algorithm performs significantly better as compared to sign-based partitioning for all generated hypergraphs.

The proposed algorithm is compared to the conventional sign-based partitioning using the Fiedler vector computed from the Laplacian tensor of the hypergraph. It should be noted that computation of tensor eigenvalues in NP-hard and cannot be approximated unless P = NP [22]. A histogram plot summarizing the results is shown in Fig 6.

**Fig 7:**
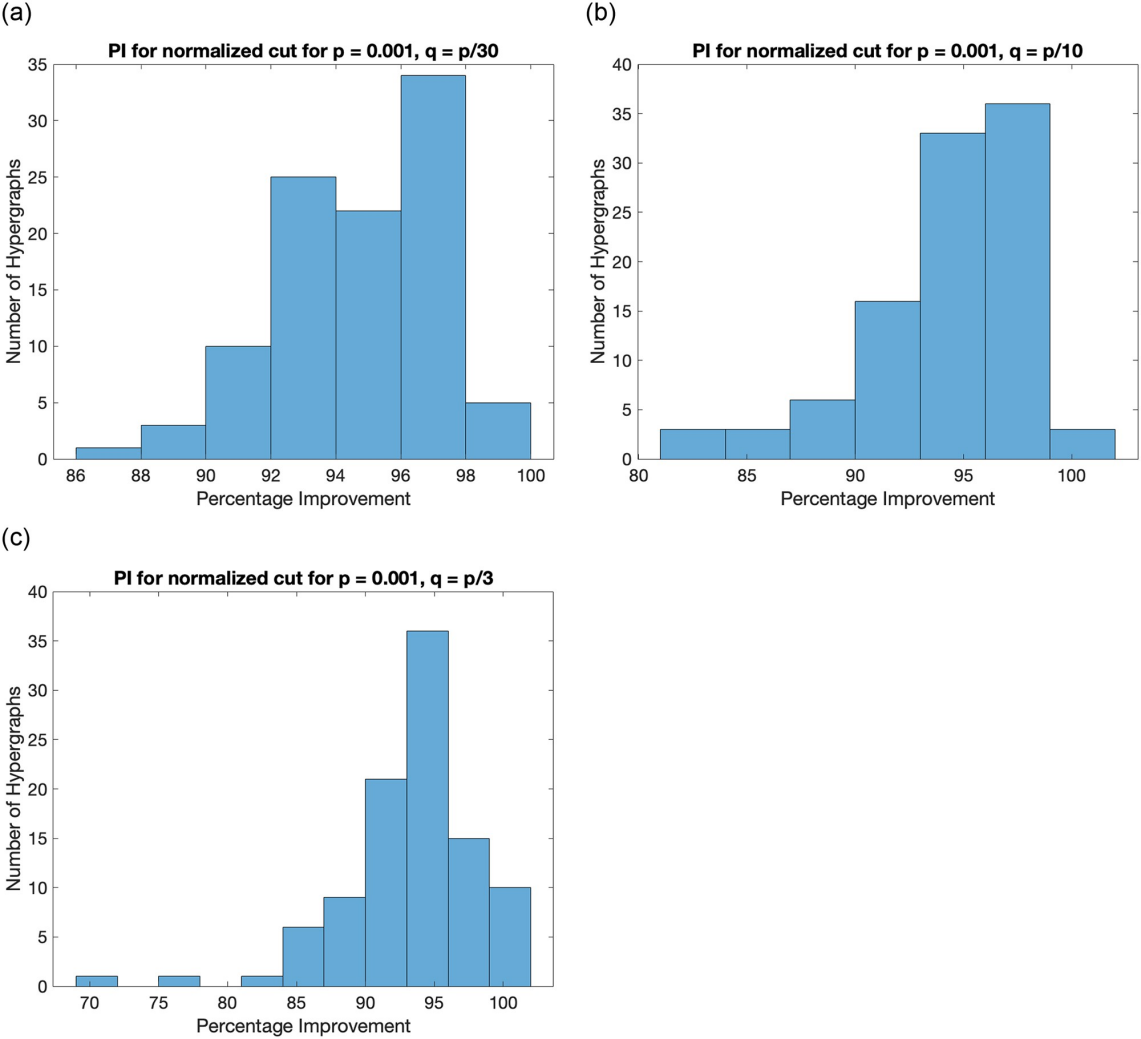
Histogram plot for percentage improvement by the proposed method for normalized cut value on hypergraphs generated by the SBM for different values of *q*. It shows that the proposed algorithm performs significantly better as compared to sign-based partitioning for all generated hypergraphs.

We perform a similar analysis on the comparison of proposed algorithm and sign-based partitioning using Fiedler vector of normalized Laplacian tensor of the hypergraph. This is done to study the behaviour of algorithm for normalized cut defined in Eq (16). The histogram plot is presented in Fig 7.

It can be observed that the proposed algorithm has improved the ratio-cut value (defined in Eq (15)) and normalized cut value (defined in Eq (16)) significantly as compared to the traditional sign-based partitioning. This is primarily because cutting a few hyperedges does not necessarily produce only two components, unlike the case of graphs. For example, if we cut a hyperedge having 3 nodes in a hypergraph with one hyperedge only, we get 3 disconnected components, and there is no possibility of obtaining two connected components. Hence, we may get 3 connected components, even if we desired only 2 connected components.

Any partitioning algorithm producing many small connected components (like singletons) is likely to have a higher ratio-cut value. We observe that the sign-based partitioning approach using the Fiedler vector of the Laplacian tensor is more prone to producing many small connected components as compared to the results by proposed algorithms. Hence, the ratio-cut value or normalized cut value by sign-based partitioning is significantly higher.

## 5 Conclusions & future work

In this work, we propose a hypergraph partitioning algorithm using tensor eigenvalue framework which removes the hyperedges directly without performing reduction to a graph like existing methods. This was done by using the novel “hyperedge score” metric. To do this, we extended the definition of ratio-cut and normalized cut from graphs to hypergraph and showed the equivalence of relaxed optimization problem to a tensor eigenvalue problem. Further, we derived a tighter upper bound for the approximation of normalized-cut problem. The future directions of this work is along the lines of similar analysis for non-uniform and directed hypergraphs.

## Supporting information

S1 File (ZIP)
