# Supplementary material for: Hypergraph partitioning using tensor eigenvalue decomposition
Source: PLoS One. 2023 Jul 21;18(7):e0288457. doi: 10.1371/journal.pone.0288457 (PMC10361499; doi:10.1371/journal.pone.0288457)
Supplement: S1 File — (ZIP) [file pone.0288457.s001.zip › hypart_SI_jul8.pdf]

Supporting information for Hypergraph Partitioning using Tensor Eigenvalue Decomposition

A Examples & Numerical Details of Experiments

A.1 Hypergraph reduction to same graph

Various hypergraph reduction methods have been summarized in Agarwal et al. [1]. One of the prevalent approaches is

$$\mathbf{A}_r = \mathbf{HWH}^T - \mathbf{D} \tag{1}$$

It should be noticed that these reduction methods are a non-unique mapping from hypergraph to adjacency matrix. This implies that there could be multiple different hypergraphs which reduce to same graph. For example, the clique reduction approach reduces the four-uniform hypergraph and the three-uniform hypergraph to the same graph as shown in Figure 1 below:

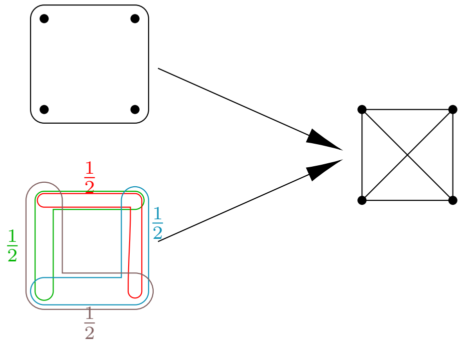

Figure 1: Two Hypergraphs reducing to same Graph

This non-uniqueness property of hypergraph reduction method plays a very crucial role in the task of hypergraph partitioning. The reduced hypergraph has lost the information about the original hypergraph structure. So there is no assurance of any analysis on reduced hypergraph to deliver correct results for the original hypergraph. To avoid the loss of information in the reduction step, we utilize the tensor-based representation of hypergraphs as shown in the next example.

A.2 Representation of Hypergraphs

Consider a $4-$uniform hypergraph $G(V, E)$, where the set of vertices and hyperedges are defined by

$$V = \{1, 2, 3, 4, 5\}$$
$$E = \{\{1, 2, 3, 4\}, \{2, 3, 4, 5\}, \{1, 2, 3, 5\}\}$$

The adjacency tensor for the above hypergraph is denoted by $\mathcal{A}$ and has dimensions $5 \times 5 \times 5 \times 5$. It should be noted that the cardinality (k) of all 3 hyperedges is 4 . The elements of $\mathcal{A}$ are denoted by a_{i_1,i_2,i_3,i_4}, where $1 \leq i_k \leq 5$. It should be noted that $\mathcal{A}$ contains $n^k = 5^4$ elements but only $m \times k! = 3 \times 4! = 72$ elements have non-zero entries. The elements corresponding to first hyperedge are

described by

$$\begin{aligned}
&a_{1234} = a_{1243} = a_{1324} = a_{1342} = a_{1423} = a_{1432} \\
=&a_{2134} = a_{2143} = a_{2314} = a_{2341} = a_{2413} = a_{2431} \\
=&a_{3214} = a_{3241} = a_{3124} = a_{3142} = a_{3421} = a_{3412} \\
=&a_{4231} = a_{4213} = a_{4321} = a_{4312} = a_{4123} = a_{4132} = c
\end{aligned}$$

where $c = \frac{1}{(k-1)!} = \frac{1}{6}$. The vertex degrees can be stored in a tensor of dimension $5 \times 5 \times 5 \times 5$ with its diagonal elements being $d(v) = \begin{bmatrix} 2 & 3 & 3 & 2 & 2 \end{bmatrix}$. The tensor Laplacian has dimension of $5 \times 5 \times 5 \times 5$ and its entries are given by:

$$l_{i_1 i_2 i_3 i_4} = \begin{cases} -\frac{1}{6} & \text{if } (i_1, i_2, i_3, i_4) = \{e_j\}, \quad j = \{1, 2, 3\} \\ d(v_i) & \text{if } i_1 = i_2 = i_3 = i_4 = i \\ 0 & \text{otherwise} \end{cases} \tag{2}$$

This example shows the procedure to construct the adjacency and Laplacian tensor for any k-uniform hypergraph.

A.3 Partition Cost

First we show the cut cost derived from graph obtained from reduced hypergraph shown in Figure 1 of main manuscript. The partitions are given to be $C_1 = \{1, 2, 3\}$, $C_2 = \{4\}$ and $C_3 = \{5\}$. The hyperedges denoted by e_2 and e_3 have to be removed for such partition, so $\partial E = \{e_2, e_3\}$. The cost for partitions can be derived as shown below :

$$\begin{aligned}
w_h(C_1) &= \sum_{e_j \in \partial E} |C_1 \cap e_j| w_{e_j} \\
&= |C_1 \cap e_2| w_{e_2} + |C_1 \cap e_3| w_{e_3} \\
&= 2w_{e_2} + w_{e_3}
\end{aligned} \tag{3}$$

The cost of other partitions can be derived in similar fashion and are observed to be $w_h(C_2) = w_2 + w_3$ and $w_h(C_3) = w_3$.

Further, we compute the cut cost for reduced hypergraph. The edges between node i and j is named as e_{ij} and the corresponding weight is denoted by $w_{e_{ij}}$. The edges denoted by $e_{24}, e_{34}, e_{35}, e_{45}$ have to be removed for arriving at the desired partition, So $\partial E_g = \{e_{24}, e_{34}, e_{35}, e_{45}\}$. The cut cost for such partitioning is given by:

$$\begin{aligned}
w_g(C_1) = &\sum_{e_{ij} \in \partial E_g} |C_1 \cap e_{ij}| w_{e_{ij}} \\
&= |C_1 \cap e_{24}| w_{e_{24}} + |C_1 \cap e_{34}| w_{e_{34}} + |C_1 \cap e_{35}| w_{e_{35}} + |C_1 \cap e_{45}| w_{e_{45}} \\
&= w_{e_{24}} + w_{e_{34}} + w_{e_{35}} + 0 \\
&= w_{e_2} + (w_{e_2} + w_{e_3}) + w_{e_3} = 2(w_{e_2} + w_{e_3})
\end{aligned} \tag{4}$$

The cut cost for other partitions can be calculated as $w_g(C_2) = 2(w_{e_2} + w_{e_3})$ and $w_g(C_3) = 2w_{e_3}$.

It can be easily noticed that the cut cost for both the cases are not equal. On further inspection, we infer $w_g(C_i) = 2w_h(C_i)$ for $i = \{2, 3\}$, which means the cut cost for partitions C_2 and C_3 in reduced hypergraph are just a scaled version of costs involved in original hypergraph. The same relation doesn't hold for partition C_1 due to the presence of the term $|C_i \cap e_j|$.

A.4 Numerical details of Example 2

The Fiedler eigenvalue for this hypergraph is reported to be 0.0372 and the corresponding eigenvector is given by:

$$\mathbf{f} = \begin{bmatrix}0.34 & 0.21 & 0.18 & 0.11 & -0.07 & 0.05 & 0.12 & 0.38 & 0.39 & 0.39 & 0.38 & 0.39\end{bmatrix}^T \quad (5)$$

It is clear that the classical spectral approach does not produce the optimal partitions and hence we compute the hyperedge scores as prescribed by the proposed algorithm. The hyperedge scores are presented in Table 1.

Table 1: Hyperedge-Score for Example 2

Hyperedge	Score
$\{1,2,3\}$	0.0170
$\{4,5,6\}$	0.0060
$\{2,3,4\}$	0.0041
$\{3,4,7\}$	0.0036
$\{1,8,11\}$	0.0033
$\{4,6,7\}$	0.0024
$\{8,9,10\}$	0.0005
$\{10,11,12\}$	0.0005
$\{9,10,12\}$	0

We observe the maximum score of 0.017 for the hyperedge $\{1,2,3\}$ and hence cut it to obtain the optimal partitions $A_1 = \{2,3,4,5,6,7\}$ and $\bar{A}_1$.

References

[1] Agarwal S, Branson K, Belongie S. Higher order learning with graphs. In: Proceedings of the 23rd International Conference on Machine learning. ACM; 2006. p. 17–24.
